# Supplementary material for: miR-28-based combination therapy impairs aggressive B cell lymphoma growth by rewiring DNA replication
Source: Cell Death Dis. 2023 Oct 18;14(10):687. doi: 10.1038/s41419-023-06178-0 (PMC10585006; doi:10.1038/s41419-023-06178-0)
Supplement: Supplementary file 1 — Supplementary Figures with figure legeds [file 41419_2023_6178_MOESM1_ESM.pdf]

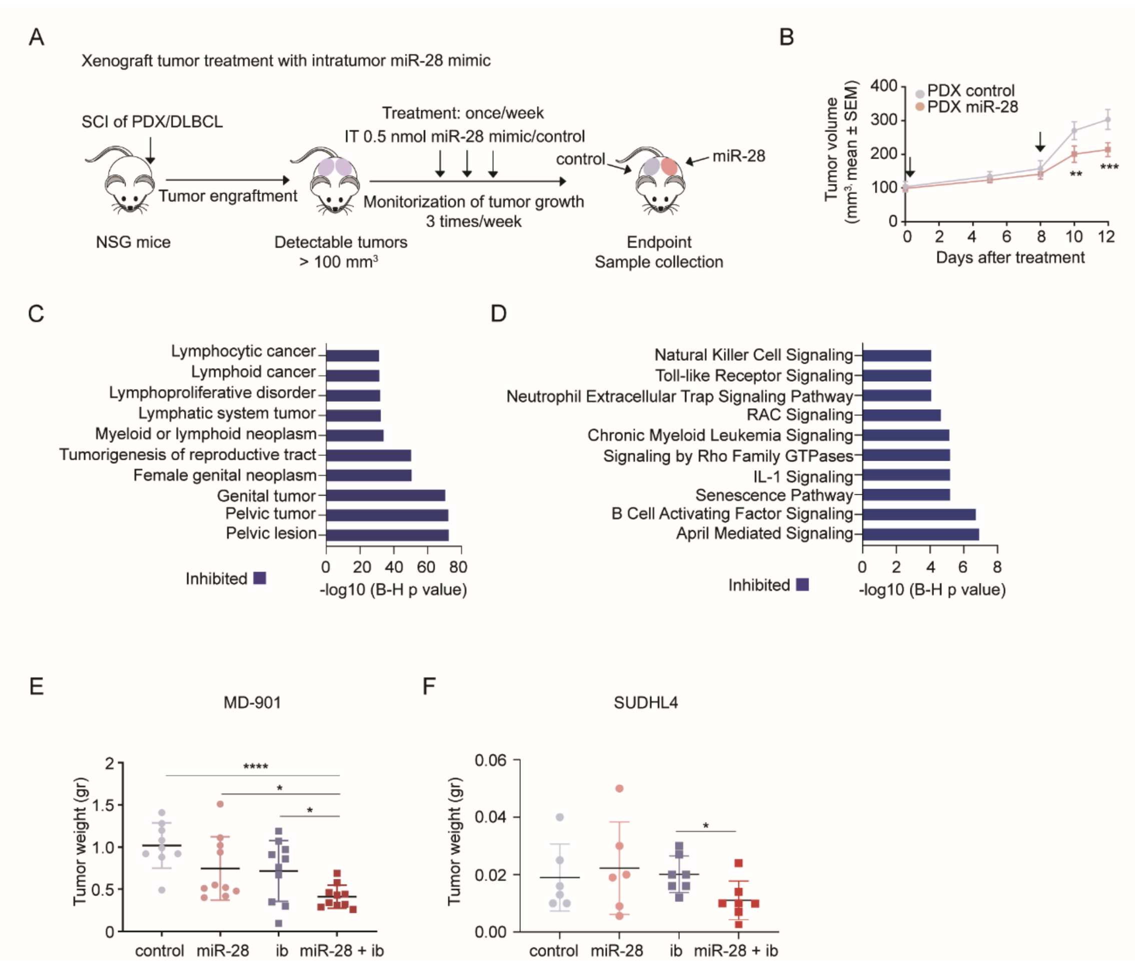

Figure S1. miR-28 based therapies assessed in DLBCL xenograft models. (A, B) DLBCL xenograft models treated by intratumoral injection of miR-28 mimics. **(A)** Experimental model. DLBCL cell lines or ABC-DLBCL PDX (PRoXe DFBL-18689-V2) cells were injected subcutaneously into NSG mice. When tumors were detectable (>150 mm^3 for DLBCL cell lines and > 100 mm^3 for ABC-DLBCL PDX), mice were given intratumoral injections of 0.5 nmol miRNA mimics: miR-28 (pink) or control (gray). Tumor volume was measured 3 times per week. **(B)** Volume of ABC-DLBCL PDX tumors after treatment, calculated as mean volume ± SEM (3 independent experiments, n=22 control-treated tumors, n=21 miR-28-treated tumors). **P<0.01, ***P<0.001, linear mixed model. **(C-D)** RNA-seq analysis of pTRIPZ-scramble or miR-28 MD-901 tumors 8 days after doxycycline administration (n=3 miR-28, n=3 control).

The graphs show Benjamini-Hochberg (B-H) adjusted p-values for comparative IPA of differentially expressed genes (DEGs). Pathway activity prediction is shown with a color code (blue, inhibited by miR-28). P-values were corrected for multiple testing using the Benjamini-Hochberg (B–H) false discovery rate. **(C)** The top 10 significantly inhibited Diseases & Functions Pathways. **(D)** The top 10 significantly inhibited Canonical Pathways. **(E-F)** NSG mice were SC injected with MD-901 or SUDHL4 cells transduced with doxycycline-inducible pTRIPZ-miR-28 or pTRIPZ-scramble. Mice were treated with daily administrations of 40 mg/kg (MD-901) or 20 mg/kg (SUDHL4) ibrutinib or vehicle and doxycycline in the drinking water. Tumor weight was measured at endpoint in the four treatment conditions (control, miR-28, ibrutinib, miR-28+ibrutinib) in MD-901 (n= 9-10 tumors per group) **(E)** and SUDHL4 (n= 6-7 tumors per group) **(F)**. *P<0.05 ****P<0.0001, unpaired *t* test. Error bars denote SD.

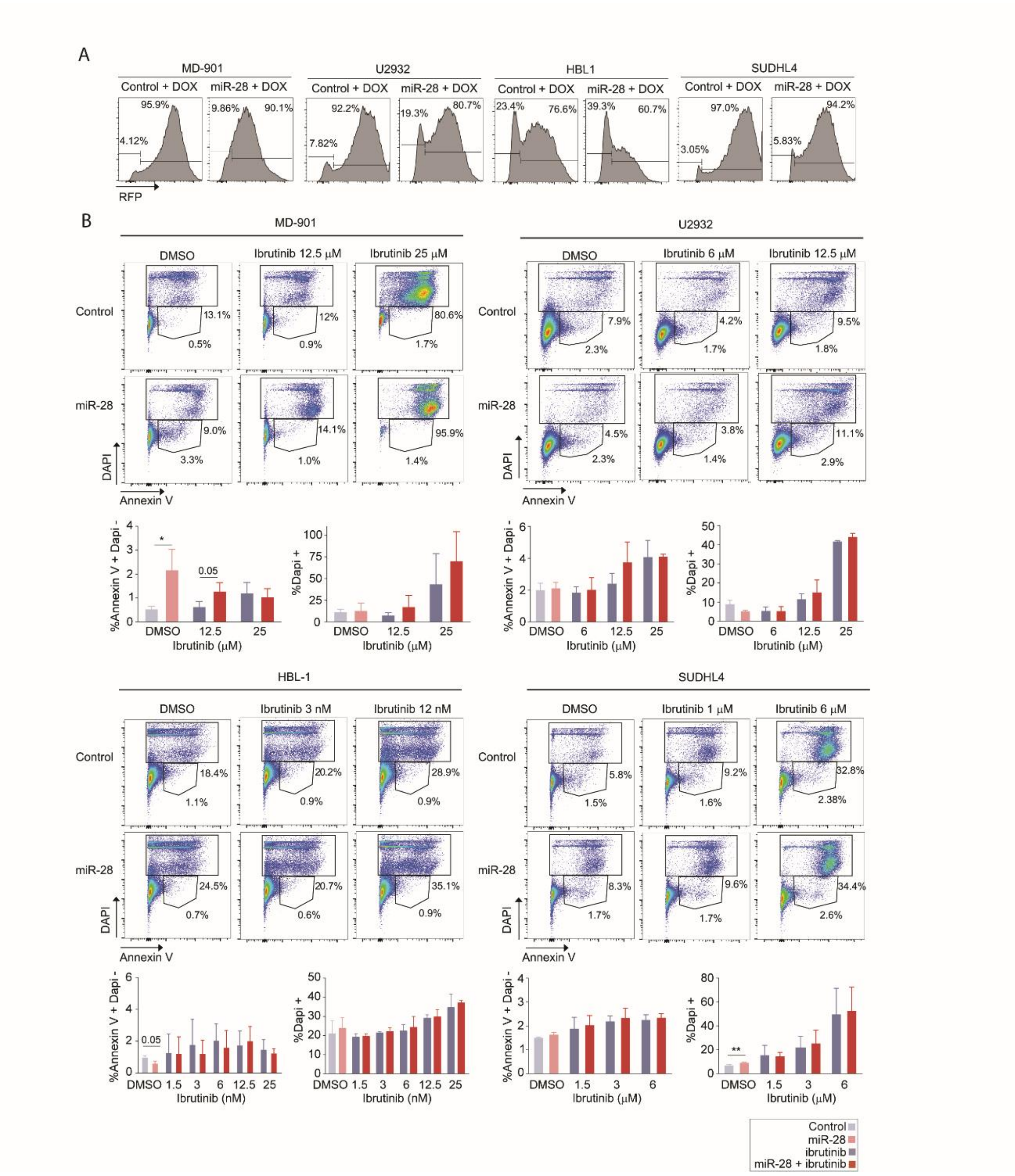

Figure S2. RFP expression and cell death analysis of DLBCL cell lines after miR-28+ibrutinib combined treatment. (A) Representative flow cytometry plots of RFP expression by pTRIPZ-miR-28– or pTRIPZ-scramble–transduced DLBCL cell lines induced with doxycycline for 3 days. **(B)** pTRIPZ-miR-28– or pTRIPZ-scramble–transduced DLBCL cell lines were treated with serial dilutions of ibrutinib in the presence of doxycycline. The percentage of apoptotic (Annexin V^{+} $Dapi^{-}$) or dead ($Dapi^{+}$) cells was analyzed at day 3 by FACS with Annexin V and Dapi staining. The bar plots show

quantification of cell death in response to control (light gray), miR-28 (light pink), ibrutinib (dark gray) and miR-28+ibrutinib combined treatment (dark red) in MD-901 (n=3), U2932 (n=2), HBL1 (n=2) and SUDHL4 (n=2). Error bars denote SD of n independent experiments. *P<0.05, **P<0.01, unpaired *t* test.

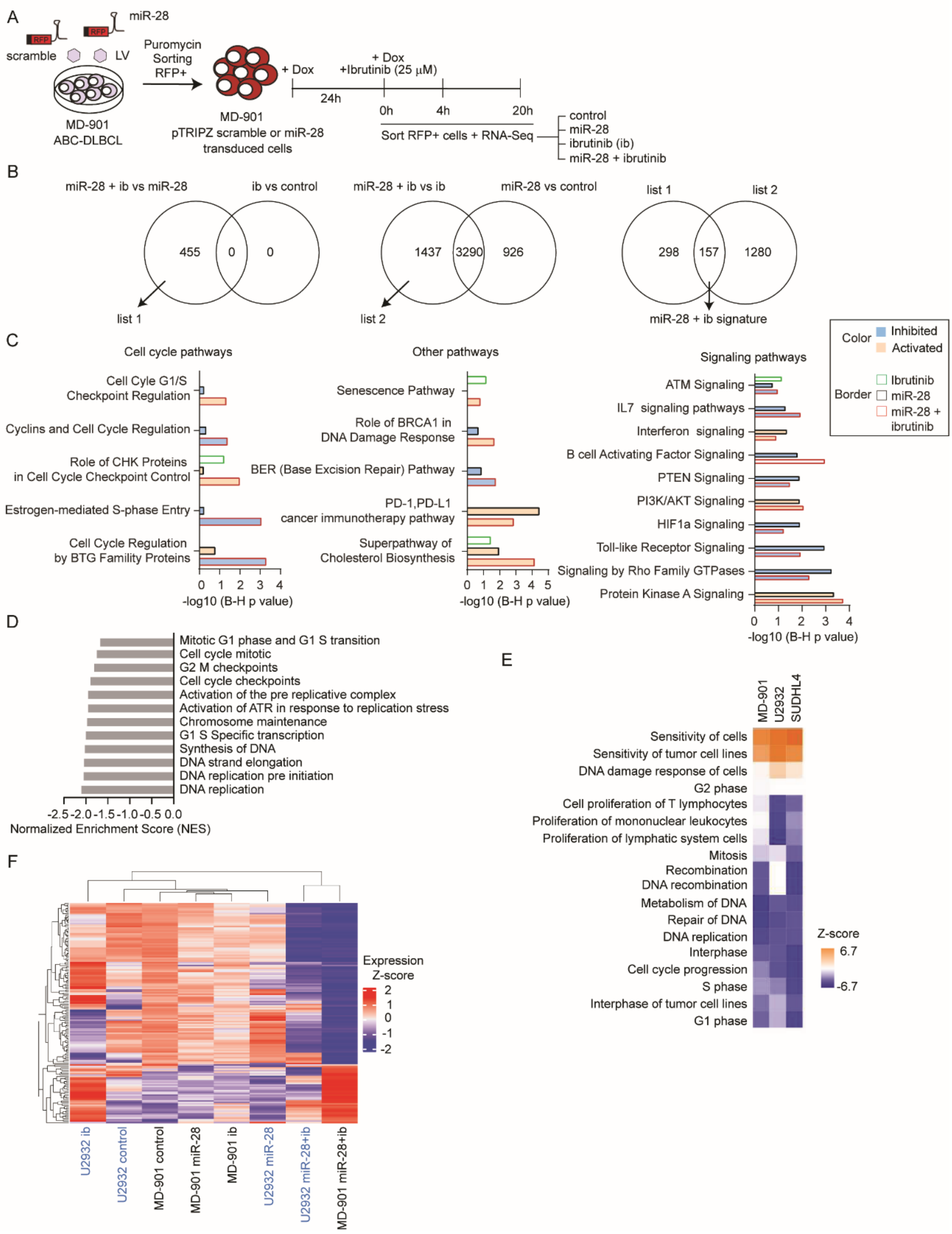

Figure S3. Effect of miR-28+ibrutinib combined treatment on DLBCL transcriptome. (A) Experimental setup. pTRIPZ-miR-28– or pTRIPZ-scramble–transduced MD-901 cells were selected in the presence of puromycin, and RFP^{+} cells

were isolated by FACS. Cells were induced with doxycycline for 24 hours before treatment with ibrutinib (25 μM). RNA-seq was performed in RFP^+ cells isolated by FACS after 0, 4 and 20 hours of ibrutinib treatment. **(B)** Analysis used to identify the miR-28+ib signature in MD-901 treated cells. **(C)** Bar plots showing Benjamini-Hochberg (B-H) adjusted p-values for comparative IPA of differentially expressed genes (DEGs) in response to ibrutinib (green border), miR-28 (black border), and miR-28+ibrutinib (red border) compared with control. Terms are colored according to the Z-score (pathway activation prediction): positive values (activation, in orange) and negative values (inhibition, in blue). **(D)** Bar graph showing Normalized Enrichment Scores (NES) for the GSEA of miR-28+ibrutinib combined treatment versus control. **(E)** Heatmap showing Z-score values of pathways identified by comparative IPA of DEGs in response to miR-28+ibrutinib treatment in MD-901, U2932 and SUDHL4 cells after 20 hours of treatment **(F)** Hierarchical clustering heatmap showing Z-score expression values of the 157 miR-28+ib signature genes at 20 hours in the four treatment conditions (control, miR-28, ibrutinib and miR-28+ibrutinib) in MD-901 and U2932 cells.

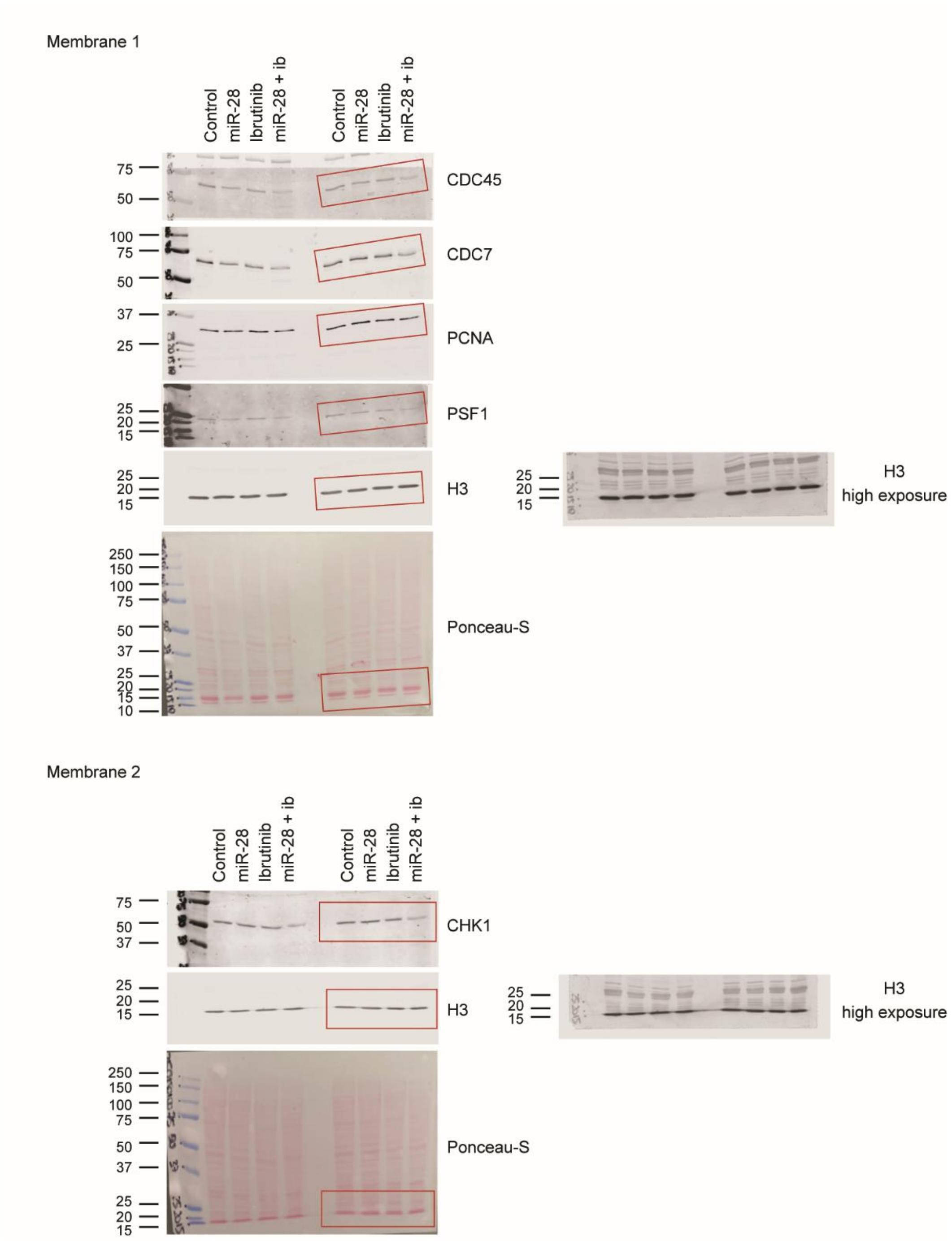

Figure S4. miR-28+ibrutinib combined treatment downregulates the expression of DNA replication proteins. Uncropped western blot membranes (figure 3F) of selected replication proteins analyzed in doxycycline-induced pTRIPZ-miR-28– or pTRIPZ-scramble–transduced MD-901 cells after treatment for 20 hours with 25 μM ibrutinib or

DMSO. Histone H3 (H3) was used as loading control. H3 high exposure images are displayed to show the marker.
